# Alternative substrate-bound conformation of bacterial solute-binding protein involved in the import of mammalian host glycosaminoglycans

**DOI:** 10.1038/s41598-017-16801-8

**Published:** 2017-12-05

**Authors:** Sayoko Oiki, Reiko Kamochi, Bunzo Mikami, Kousaku Murata, Wataru Hashimoto

**Affiliations:** 1 Laboratory of Basic and Applied Molecular Biotechnology, Division of Food Science and Biotechnology, Graduate School of Agriculture, Kyoto University, Uji, Kyoto, 611-0011 Japan; 2 Laboratory of Basic and Applied Molecular Biotechnology, Department of Food Science and Biotechnology, Faculty of Agriculture, Kyoto University, Uji, Kyoto, 611-0011 Japan; 3 Laboratory of Applied Structural Biology, Division of Applied Life Sciences, Graduate School of Agriculture, Kyoto University, Uji, Kyoto, 611-0011 Japan; 4 Laboratory of Food Microbiology, Department of Life Science, Faculty of Science and Engineering, Setsunan University, Neyagawa, Osaka, 572-8508 Japan

## Abstract

Glycosaminoglycans (GAGs), constituted by repeating uronate and amino sugar units, are major components of mammalian extracellular matrices. Some indigenous and pathogenic bacteria target GAGs for colonization to and/or infection of host mammalian cells. In Gram-negative pathogenic *Streptobacillus moniliformis*, the solute-binding protein (Smon0123)-dependent ATP-binding cassette (ABC) transporter incorporates unsaturated GAG disaccharides into the cytoplasm after depolymerization by polysaccharide lyase. Smon0123, composed of N and C domains, adopts either a substrate-free open or a substrate-bound closed form by approaching two domains at 47° in comparison with the open form. Here we show an alternative 39°-closed conformation of Smon0123 bound to unsaturated chondroitin disaccharide sulfated at the C-4 and C-6 positions of *N*-acetyl-d-galactosamine residue (CΔ4S6S). In CΔ4S6S-bound Smon0123, Arg204 and Lys210 around the two sulfate groups were located at different positions from those at other substrate-bound 47°-closed conformations. Therefore, the two sulfate groups in CΔ4S6S shifted substrate-binding residue arrangements, causing dynamic conformational change. Smon0123 showed less affinity with CΔ4S6S than with non-sulfated and monosulfated substrates. ATPase activity of the Smon0123-dependent ABC transporter in the presence of CΔ4S6S was lower than that in the presence of other unsaturated chondroitin disaccharides, suggesting that CΔ4S6S-bound Smon0123 was unpreferable for docking with the ABC transporter.

## Introduction

Extracellular matrices are fibrillar network structures that lie under epithelial tissue cells and surround connective tissue cells^1^. Almost all mammals have extracellular matrices to provide mechanical strength to tissues and to regulate cell proliferation and differentiation^2^. Glycosaminoglycans (GAGs) are acidic heteropolysaccharides that comprise disaccharide-repeating units of uronate and amino sugar. GAGs are major components of mammalian extracellular matrices and are divided into the following three major groups: hyaluronan, chondroitin sulfate/dermatan sulfate, and heparin/heparan sulfate on the basis of the constitutional unit, sulfation level, and glycoside linkage mode^3^.

**Figure 1:**
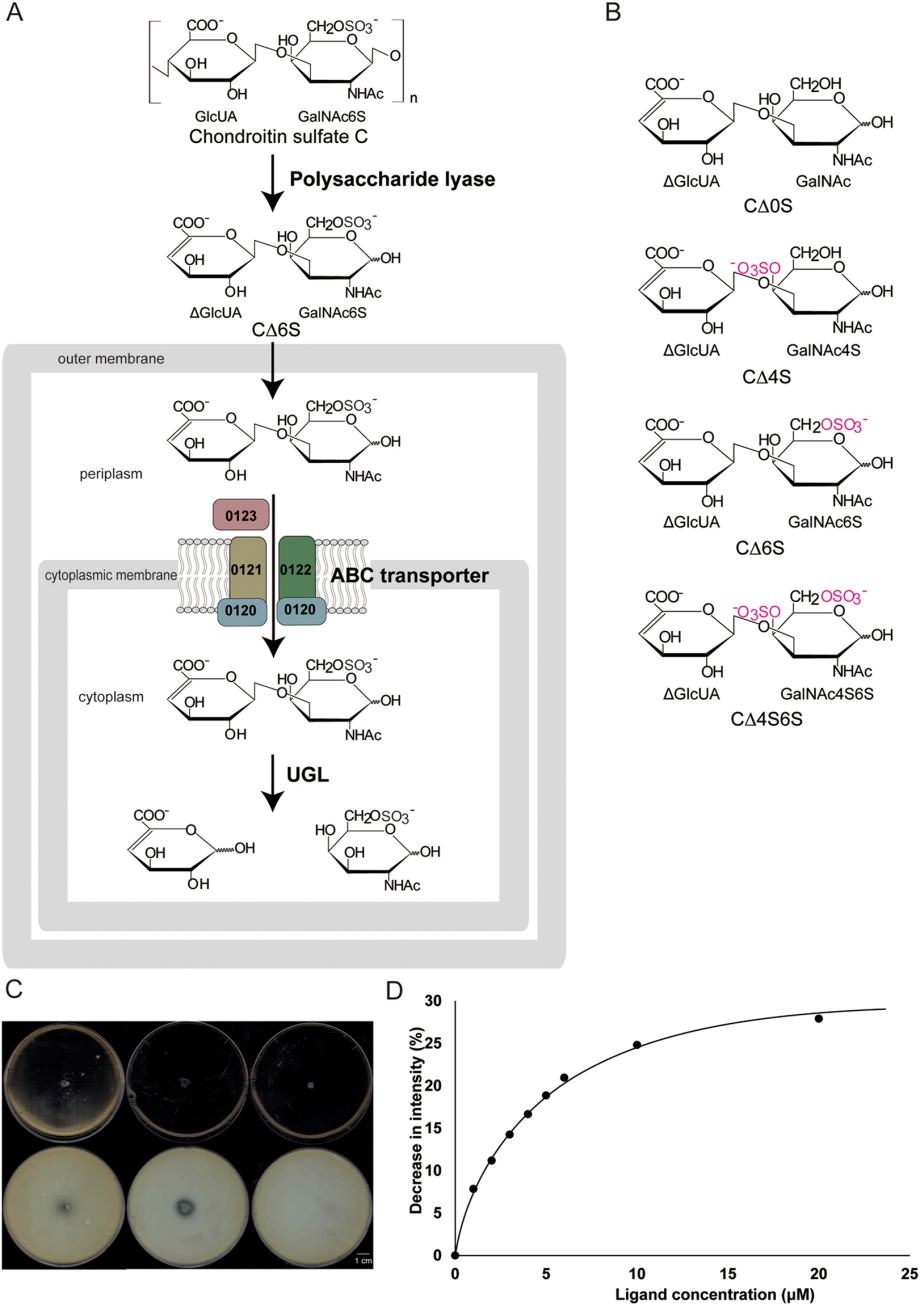
Gram-negative *Streptobacillus* GAG import system. (**A**) A model for GAG (chondroitin sulfate C) import. Polysaccharide GAGs are depolymerized to unsaturated disaccharides by extracellular or cell-surface polysaccharide lyases. Unsaturated GAG disaccharides are incorporated in the cytoplasm by the ABC transporter (Smon0121-Smon0122/Smon0120-Smon0120) through the periplasmic solute-binding protein (Smon0123). Incorporated disaccharides are degraded to monosaccharides by cytoplasmic UGL and are metabolized by some enzymes. (**B**) Structural formulas of unsaturated chondroitin disaccharides. (**C**) The degradation of chondroitin sulfate A. Upper, before adding acetic acid; and lower, after adding acetic acid. Left, *S. moniliformis*; middle, *Pedobacter heparinus* as the positive control; and right, *Escherichia coli* as the negative control. (**D**) Decrease in intensity of Smon0123 by adding increasing CΔ4S6S was plotted after a modification based on the volume change in the cuvette.

Numerous bacteria interact with GAGs for recognizing and binding to their target host cells^4^. Among some of these bacteria, extracellular or cell surface polysaccharide lyases depolymerize GAGs to obtain unsaturated GAG disaccharides with C = C double bonds at the non-reducing terminus of uronate via β-elimination reactions^5^. The resultant GAG disaccharides are degraded to monosaccharides (unsaturated uronate and amino sugar) by unsaturated glucuronyl hydrolase (UGL) via the hydration of C = C double bonds in the cytoplasm^6^. Metabolic enzymes, i.e. isomerase, NADH-dependent reductase, kinase, and aldolase, subsequently metabolize the unsaturated uronate to pyruvate and glyceraldehyde-3-phosphate^7^. The genes encoding these enzymes that are essential for depolymerization, degradation, and metabolism of GAGs assemble a cluster in the bacterial genome. We have recently identified a solute-binding protein-dependent ATP-binding cassette (ABC) transporter to be the first bacterial import system of both sulfated and non-sulfated GAG disaccharides in pathogenic *Streptobacillus moniliformis* ^8^. *S. moniliformis*, a causative organism of rat bite fever, is characterized by relapsing fever, rash, and migratory polyarthralgias^9^. The solute-binding protein (Smon0123) captures GAG disaccharides in the periplasm and delivers them to the ABC transporter, followed by their import in the cytoplasm (Fig. 1A). The ABC transporter comprises membrane-spanning proteins (Smon0121-Smon0122) as heterodimers and ATPase domains (Smon0120-Smon0120) as homodimers. The solute-binding protein-dependent ABC transporter is also encoded in the GAG genetic cluster. On the basis of the primary structure, the *S. moniliformis* ABC transporter system (Smon0123/Smon0121-Smon0122/Smon0120-Smon0120) is similar to the bacterial alginate ABC transporter system (AlgQ2/AlgM1-AlgM2/AlgS-AlgS), the structure of which has been determined in our previous paper^10^.

Chondroitin sulfate comprises d-glucuronic acid (GlcUA)/l-iduronic acid (IdoUA) and *N*-acetyl-d-galactosamine (GalNAc)^11^, which are linked by a 1,3-glycoside bond. Depending on the position and/or level of the sulfate groups, chondroitin sulfate is divided to several groups such as chondroitin sulfates A and B with a sulfate group at the C-4 position of GalNAc (chondroitin sulfates A and B contain GlcUA and IdoUA, respectively); chondroitin sulfate C with a sulfate group at the C-6 position of GalNAc; chondroitin sulfate D with two sulfate groups at the C-2 position of GlcUA and the C-6 position of GalNAc; and chondroitin sulfate E sulfated at the C-4 and C-6 positions of GalNAc^12^. Unsaturated chondroitin disaccharides are commonly termed as follows: CΔ0S, non-sulfated; CΔ4S, sulfated at the C-4 position of GalNAc; CΔ6S, sulfated at the C-6 position of GalNAc; and CΔ4S6S, sulfated at the C-4 and C-6 positions of GalNAc^13^ (Fig. 1B).

ABC transporters in Gram-negative and Gram-positive bacteria generally receive substrates from solute-binding proteins and incorporate them in the cytoplasm^14–16^. Because solute-binding proteins have a high affinity with specific substrates [The dissociation constant (*K*_d_ value) = 0.01–10 μM], these binding proteins play important roles in determining a strict substrate specificity for import^17^. This study assesses the characteristic conformational change of CΔ4S6S-bound Smon0123, the structural determinants of Smon0123 for substrate-dependent conformations, and the modeling of Smon0123/Smon0121-Smon0122/Smon0120-Smon0120 on the basis of the alginate ABC transporter system (AlgQ2/AlgM1-AlgM2/AlgS-AlgS). These findings provide structural and functional insights for further understanding substrate recognition and import and for developing inhibitors for pathogenic bacteria.

## Results

### Degradation of chondroitin sulfate A by *S. moniliformis*

Degradation of chondroitin sulfate A by *S. moniliformis* DSM 12112 was investigated using a simple plate method^18^. Bacterial cells were cultured on a plate that contained bovine serum albumin (BSA) and chondroitin sulfate A. The non-degraded GAGs aggregated with BSA to form white precipitates in the presence of acetic acid, whereas degraded GAGs showed clear zoned halos. This simple method is feasible for detecting GAG degradation by various bacteria. In the plate, clear halo zones were observed around *S. moniliformis* cells, indicating that this bacterium could degrade chondroitin sulfate A (Fig. 1C), as well as hyaluronan and chondroitin sulfate C^8^. We have previously demonstrated that the ABC transporter incorporated GAG disaccharides derived from hyaluronan (ΔHA, unsaturated hyaluronan disaccharide) and chondroitin sulfate (CΔ0S, CΔ4S, and CΔ6S)^8^. This substrate specificity of the ABC transporter suggests that *S. moniliformis* degrades other chondroitin sulfates that have two sulfate groups in their constitutional units, such as chondroitin sulfates D and E. The plate assay using abundant GAGs was not attempted because chondroitin sulfates D and E are expensive.

### Affinity of Smon0123 with chondroitin disaccharides having two sulfate groups

To investigate the interaction between Smon0123 and CΔ4S6S, the fluorescence intensity of Smon0123, which was derived from tryptophan residues, was measured in the presence of CΔ4S6S (Fig. 1D). The *K*_d_ value was determined from the plot of the ratio of change in fluorescence intensity, which exhibits a decrease with an increasing CΔ4S6S concentration. The *K*_d_ value of Smon0123 for CΔ4S6S was determined to be 3.86 ± 0.243 μM, indicating lower affinity than that for other substrates (CΔ0S, 0.635 ± 0.122 μM; CΔ4S, 1.6 ± 0.231 μM; and CΔ6S, 2.76 ± 0.195 μM)^8^. Together with previous results, the binding ability of Smon0123 with CΔ4S6S indicates that the binding protein prefers non-sulfated substrates to sulfated substrates.

### Structural determination of CΔ4S6S-bound Smon0123

X-ray crystallography enables the directly demonstration of the binding mode of Smon0123 with CΔ4S6S. Because the crystals of Smon0123 with the full-length protein gave low-resolution X-ray diffraction data^8^, the N-terminal 18 and C-terminal 5 residues-truncated Smon0123 (N-18/C-5) was used for crystallization. The purified Smon0123 (N-18/C-5) was crystallized in the presence of CΔ4S6S, and the resultant crystal was suitable for determining the structure. The crystal of Smon0123 (N-18/C-5)/CΔ4S6S belongs to a space group *P*1 and has unit lattice constants of a = 49.7, b = 69.2, c = 166 Å, α = 89.9, β = 90.0, and γ = 90.0°. Because there was a possibility that the crystal belonged to monoclinic or orthorhombic space group, we reexamined the extinction rule and tried to perform refinement under condition of monoclinic or orthorhombic space group. As a result, the correct space group was found to be *P*1 and insufficient decrease in *R*_work_ was observed in refinement under condition of monoclinic or orthorhombic space group. The structure of Smon0123 (N-18/C-5)/CΔ0S (PDB ID, 5GUB) was used as a search model for molecular replacement. After the molecular replacement, rigid body refinement was performed by using N- and C-terminal domains of Smon0123 (N-18/C-5)/CΔ0S. The refined model contained four monomers in an asymmetric unit while the biological unit was monomer. The final model of the complex was refined to *R*_work_ of 17.3% and *R*_free_ of 21.6% up to a resolution of 1.95 Å. Ramachandran plot analysis revealed that 97.9% of residues were in the favored regions and 2.12% were in the additional allowed regions. Statistics of diffraction and refinement data are shown in Table S1.

### Overall structure and substrate-binding site of CΔ4S6S-bound Smon0123

**Figure 2:**
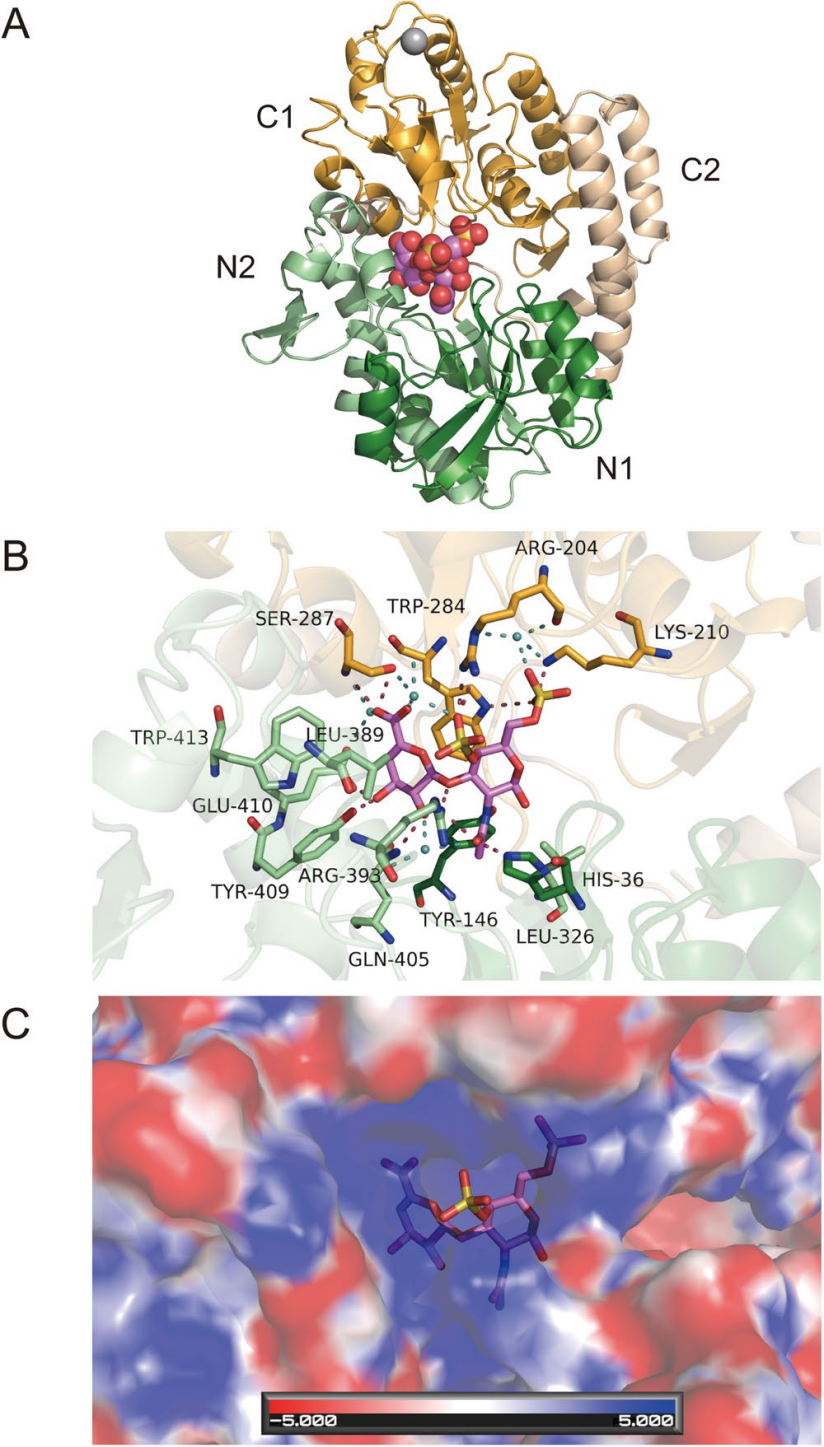
Three-dimensional structure of CΔ4S6S-bound Smon0123. Overall structure (**A**) and the binding mode of Smon0123 to CΔ4S6S (**B**). (**A**) Green, N1 subdomain; light green, N2 subdomain; orange, C1 subdomain; and light orange, C2 subdomain. The ball model shows CΔ4S6S (pink, carbon atom; red, oxygen atom; blue, nitrogen atom; and yellow, sulfur atom). Gray ball shows the calcium ion. (**B**) The pink stick model shows CΔ4S6S (pink, carbon atom; red, oxygen atom; blue, nitrogen atom; and yellow, sulfur atom). Stick models show Smon0123 amino acid residues that interact with CΔ4S6S via hydrogen bonds and/or van der Waals contacts (green/light green/orange, carbon atom; red, oxygen atom; and blue, nitrogen atom). Dark red and cyan dashed lines show direct and indirect hydrogen bonds between Smon0123 and CΔ4S6S, respectively. Small cyan balls show water molecules. (**C**) Electrostatic potential of the substrate-binding pocket. The stick model shows CΔ4S6S. Blue and red surfaces refer to positively and negatively charged spaces, respectively.

We have previously determined the four crystal structures of Smon0123 and clarified the specific interactions between the substrate and protein^8^. Thus, the conformational changes and the classification of Smon0123 in bacterial solute-binding proteins in the ABC importer system will be examined hereafter. In general, solute-binding proteins induce a conformational change via the hinge-bending motion, which is one of the two basic mechanisms of protein flexibility. The hinge-bending motion is characterized by a specific torsion angle that changes the rest of the whole proteins into a rigid body, whereas the other mechanism is shear motion, which is characterized by sliding layered structures over one another^19^. Hundreds of structures of solute-binding proteins have been previously determined using various substrates such as sugars, metal ions, amino acids, and peptides^20,21^. Although Smon0123 shows little sequence similarity with other solute-binding proteins, these constructional features are highly conserved^22,23^. Similar to other solute-binding proteins, Smon0123 has two major N and C domains that are connected via a flexible hinge and that captures the substrates via a mechanism called the “Venus’s flytrap”^24^. Each major domain is divided into two subdomains (N1-C1-N2-C2 subdomains) (Fig. 2A).

In the complex crystal structure of Smon0123 with CΔ4S6S, the substrate CΔ4S6S was bound to a cleft between the N and C domains. Far from the cleft, a calcium ion was bound to the C1 subdomain. The electron density of N-terminal 9 residues (Met1Lys19-Gly26) was invisible because of structural flexibility. Such structural features mentioned above are identical to those of other substrate-bound Smon0123 [Smon0123/CΔ0S (PDB ID: 5GUB), Smon0123/CΔ4S (PDB ID: 5GX6), and Smon0123/CΔ6S (PDB ID: 5GX7)]^8^. Smon0123 strongly recognized CΔ4S6S by hydrogen bonds and van der Waals contacts in the cleft between the domains (Fig. 2B and Table 1). The cleft had spatial allowance around the two sulfate groups of CΔ4S6S. Two positively charged residues, Arg204 and Arg393, were directly bound to the sulfate group at the C-4 position. Lys210 directly formed hydrogen bonds with the sulfate group at the C-6 position. Therefore, these basic residues were concertedly situated around the negatively charged substrate, causing a positively charged binding site (Fig. 2C). Moreover, aromatic residues such as Tyr146 and Trp284 interacted with the pyranose ring of the substrate. The number of hydrogen bonds with GalNAc4S6S was more than those with ΔGlcUA. In contrast, the number of van der Waals contacts with GalNAc4S6S was less than those with ΔGlcUA.

**Table 1:** Interactions between Smon0123 and CΔ4S6S.

Hydrogen bonds (<3.3 Å)									
Sugar	Atom	Protein	Atom	Distance (Å)	Sugar	Atom	Protein	Atom	Distance (Å)
ΔGlcUA	O2	Gln405	NE2	2.92	GalNAc4S6S	O2S	Lys210	NZ	2.84
	O3	Tyr409	OH	2.62		O2S	Trp284	NE1	3.16
	O3	Glu410	OE2	2.83		O3	Arg393	NH2	2.81
	O6A	Ser287	OG	3.12		O4S	Arg393	NE	2.73
	O6A	Ser287	N	2.93		O6S	Arg204	NH1	2.97
	O2	water5		2.65		O6S	Arg204	NH2	2.90
	O6A	water706		2.81		O7	His36	NE2	2.86
	O6B	water24		2.88		O7	Arg393	NH2	3.12
						N	Tyr146	OH	2.90
						O3S	water113		2.95
						O6S	water24		2.70
						O7	water5		2.99
**van der Waals (C-C distance < 4.5 Å)**									
**Sugar**	**Atom**	**Protein**	**Atom**	**Distance (Å)**	**Sugar**	**Atom**	**Protein**	**Atom**	**Distance (Å)**
ΔGlcUA	C1	Trp284	CD2	4.23	GalNAc4S6S	C1	Leu326	CD2	4.21
	C1	Trp284	CE2	4.37		C3	Trp284	CE2	4.06
	C1	Trp284	CE3	4.07		C3	Trp284	CZ2	3.84
	C1	Trp284	CZ3	4.00		C3	Trp284	CH2	4.23
	C1	Trp284	CZ2	4.35		C4	Trp284	CE2	4.04
	C1	Trp284	CH2	4.14		C4	Trp284	CZ2	4.28
	C1	Leu389	CD2	4.01		C5	Trp284	CE2	4.13
	C2	Leu389	CD2	4.09		C5	Trp284	CZ2	4.29
	C3	Trp284	CE3	4.25		C7	His36	CE1	4.04
	C3	Trp284	CZ3	4.09		C7	Tyr146	CE1	4.36
	C3	Glu410	CD	4.09		C7	Tyr146	CZ	4.30
	C4	Trp284	CE3	3.87		C7	Leu326	CD2	3.89
	C4	Trp284	CZ3	4.25		C8	Tyr146	CE1	3.66
	C4	Leu389	CD2	3.88		C8	Tyr146	CZ	3.64
	C4	Glu410	CD	3.99		C8	Leu326	CD2	3.78
	C5	Trp284	CB	4.30					
	C5	Trp284	CG	4.31					
	C5	Trp284	CD2	4.04					
	C5	Trp284	CE3	3.76					
	C5	Trp284	CZ3	4.39					
	C5	Leu389	CD2	3.21					
	C6	Ser287	CA	4.33					
	C6	Ser287	CB	3.53					
	C6	Trp284	CB	3.47					
	C6	Trp284	CG	3.94					
	C6	Trp284	CD2	4.14					
	C6	Trp284	CE3	4.12					
	C6	Leu389	CD2	3.48					
	C6	Trp413	CH2	4.30					

O1S-O3S and O4S-O6S indicate three oxygen atoms of the sulfate groups at the C-6 and C-4 position, respectively.

### Conformational changes by substrate binding

**Figure 3:**
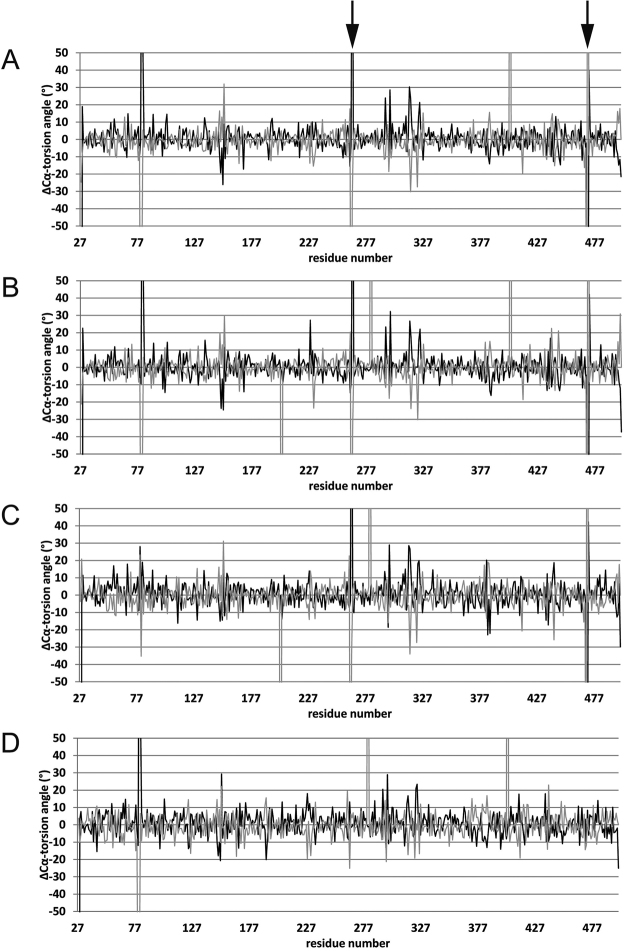
The torsion angle differences of substrate-free and substrate-bound Smon0123. Differences of Cα-torsion angles between substrate-free and CΔ0S-bound Smon0123 (**A**), CΔ4S-bound Smon0123 (**B**), CΔ6S-bound Smon0123 (**C**), and CΔ4S6S-bound Smon0123 (**D**). Black, φ plot; gray, ψ plot. Arrow shows the peaks around Gly265 and Ser470.

To draw a comparison between substrate-free Smon0123 and CΔ4S6S-bound Smon0123, the torsion angle differences of the Cα backbone between the two forms were plotted (Fig. 3). At both φ and ψ plots, structural changes occurred in the hinge region at three loops: Tyr146-Ser154 (N1-C1), Arg319-Thr324 (C1-N2), and Ala414-Ser415 (N2-C2). In particular, the only loop (Arg319-Thr324) that extended over the N and C domains showed a remarkable change, indicating that this loop pulled both the domains. Moreover, CΔ4S6S-bound Smon0123 showed a different pattern around Gly265 and Ser470 compared with other substrate (CΔ0S, CΔ4S, and CΔ6S)-bound Smon0123, suggesting a different motion of the C domain (Fig. 3, arrow).

**Figure 4:**
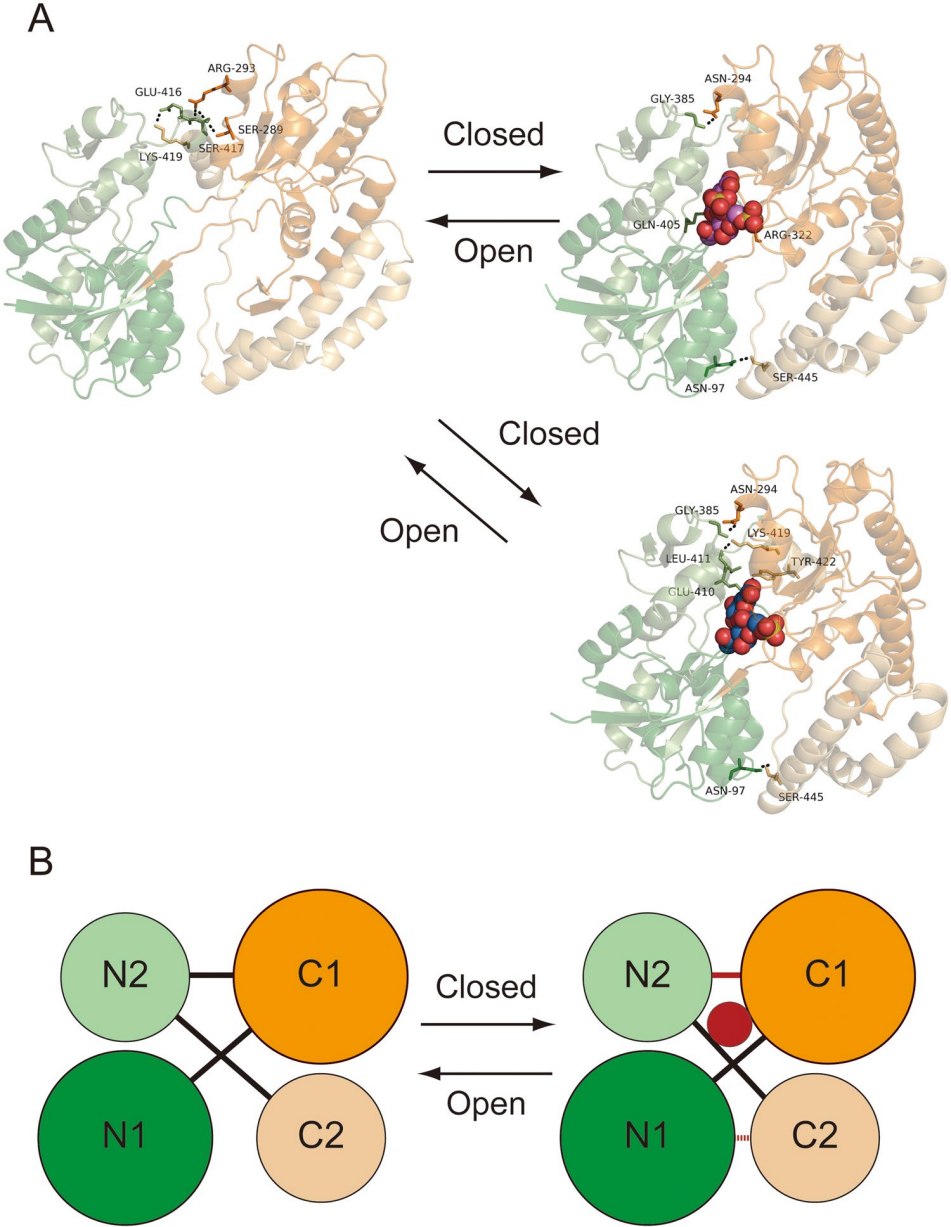
Interdomain interactions by substrate binding. Interdomain differences between substrate-free (left) and substrate-bound (right) Smon0123. Green, N1 subdomain; light green, N2 subdomain; orange, C1 subdomain; and light orange, C2 subdomain. (**A**) Upper, CΔ4S6S-bound Smon0123; and lower, CΔ6S-bound Smon0123. (**B**) Substrate binding (red circle) induces the changes of hydrogen bonds between the subdomains. In substrate-bound Smon0123, hydrogen bonds were formed between N1 and C2 subdomains (red broken line), which disappeared and were alternatively formed between N2 and C1 subdomains (red line) compared with substrate-free Smon0123.

The following differences between substrate-free and CΔ4S6S-bound Smon0123 were noted by investigating the interactions across four subdomains (N1-C1-N2-C2) (Fig. 4). (i) In substrate-bound Smon0123, Asn97 (N1 subdomain) and Ser445 (C2 subdomain) formed hydrogen bonds, which are not observed in substrate-free Smon0123. This additional bond was formed because of the close contact of N1 and C2 subdomains. (ii) Hydrogen bonds between Ser417 (N2 subdomain) and Arg293 (C1 subdomain)/Ser289 (C1 subdomain) in substrate-free Smon0123 disappeared, and alternative hydrogen bonds between Gly385 (N2 subdomain) and Asn294 (C1 subdomain) and between Gln405 (N2 subdomain) and Arg322 (C1 subdomain) were formed in substrate-bound Smon0123. (iii) The hydrogen bond between Glu416 (N2 subdomain) and Lys419 (C2 subdomain) in substrate-free Smon0123 disappeared in substrate-bound Smon0123.

Although other substrate (CΔ0S, CΔ4S, and CΔ6S)-bound Smon0123 formed hydrogen bonds between Glu410 (N2 subdomain) and Tyr422 (C2 subdomain) and between Leu411 (N2 subdomain) and Lys419 (C2 subdomain) in terms of (iii), structural features of (i) and (ii) were commonly observed in all substrate-bound Smon0123. Thus, substrate-bound Smon0123 provided additional hydrogen bonds between N1 and C2 subdomains. However, Smon0123/CΔ4S6S formed specific hydrogen bonds between N2 and C2 subdomains, which were not observed in the other substrate (CΔ0S, CΔ4S, and CΔ6S)-bound Smon0123. Substrate binding subsequently induced conformational changes between interdomain interactions, resulting in a more rigid structure than substrate-free Smon0123.

### The magnitude of hinge-bending motion

**Figure 5:**
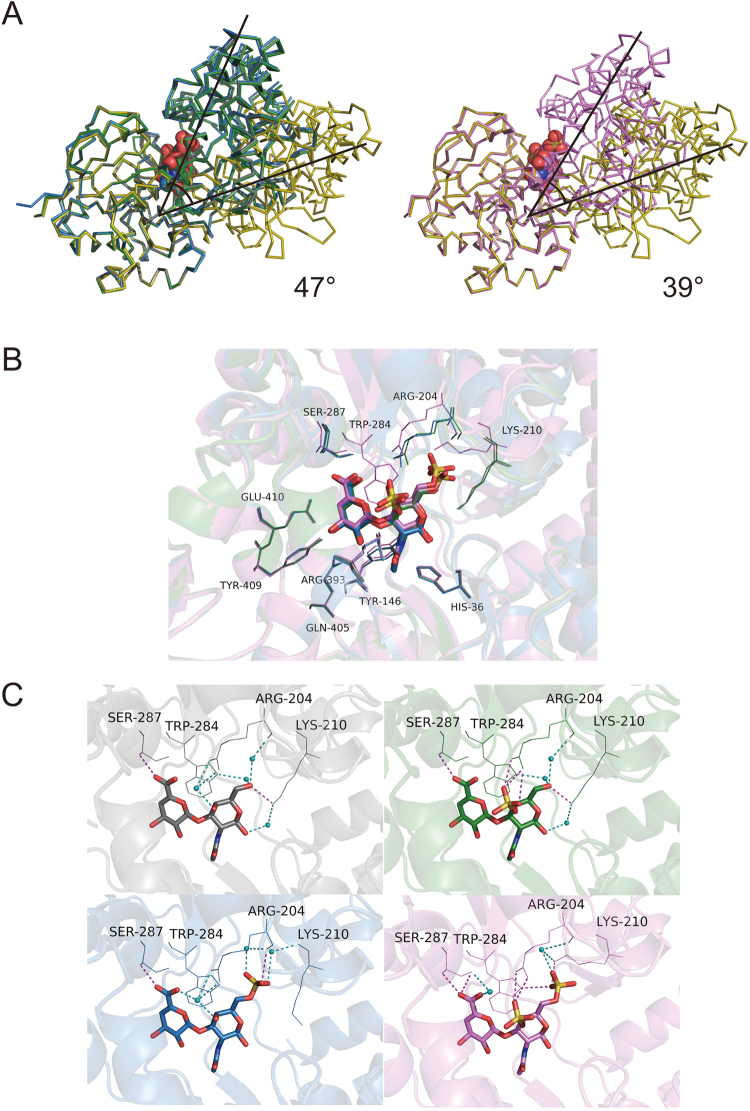
Hinge-bending motion of substrate-free and substrate-bound Smon0123. (**A**) N domains of substrate-free and substrate-bound Smon0123 were superimposed. Gray, CΔ0S-bound; green, CΔ4S-bound; blue, CΔ6S-bound; pink, CΔ4S6S-bound; and olive, substrate-free Smon0123. Ribbon models and ball models show the main chain of Smon0123 and disaccharides, respectively. Although the domains of CΔ0S-, CΔ4S-, and CΔ6S-bound Smon0123 were 47° more closed than those of substrate-free Smon0123 (left), the hinge-bending motion of CΔ4S6S-bound Smon0123 was 39° (right). (**B**) Smon0123 amino acid residues directly formed hydrogen bonds with CΔ0S (gray), CΔ4S (green), CΔ6S (blue), and CΔ4S6S (pink). (**C**) Some residues of CΔ4S6S-bound Smon0123 (pink) located in a different position from those in the other substrate-bound Smon0123 (gray, CΔ0S; green, CΔ4S; and blue, CΔ6S). The stick models show unsaturated chondroitin disaccharides, and the line models show Arg204, Lys210, Trp284, and Ser287 residues. Small cyan balls show water molecules (oxygen atoms). Magenta and cyan dashed lines show direct and indirect hydrogen bonds, respectively.

To investigate the magnitude of the hinge-bending motion, N domains of substrate-free and substrate-bound Smon0123 were superimposed, and rotation and translation of C domains were calculated from centers of gravity using the FIT program^25,26^ (Fig. 5A). The previously determined structures of substrate (CΔ0S, CΔ4S, and CΔ6S)-bound Smon0123 reveal all 47°-closed conformations compared with substrate-free Smon0123. On the other hand, the magnitude of the hinge-bending motion of the Smon0123/CΔ4S6S structure was determined to be 39°. Because the structure of each substrate is a possible factor for the difference in the magnitude, four substrate-bound Smon0123 forms (CΔ0S, CΔ4S, CΔ6S, and CΔ4S6S) were compared by superimposing their substrate-binding sites. Hence, some residues such as Arg204, Lys210, Trp284, and Ser287 in Smon0123/CΔ4S6S were located in different positions from those in other substrate-bound Smon0123 (Fig. 5B and C). Arg204 formed hydrogen bonds with each sulfate group of CΔ4S and CΔ6S, and one of the two sulfate groups (at the C-4 position) of CΔ4S6S. Lys210 made hydrogen bonds with hydroxyl groups at the C-6 position of CΔ0S and CΔ4S but not with the sulfate group of CΔ6S, whereas Lys210 formed a hydrogen bond with the sulfate group at the C-6 position of CΔ4S6S. The distance between the side chain of Lys210 in CΔ4S6S-bound Smon0123 and that in the other substrate-bound Smon0123 was approximately 6.9 Å. Trp284 formed a hydrogen bond only with the sulfate group at the C-4 position of CΔ4S6S, although all substrates (CΔ0S, CΔ4S, CΔ6S, and CΔ4S6S) were bound to Trp284 by van der Waals contacts. Ser287 formed hydrogen bonds with hydroxyl groups at the C-6 position of ΔGlcUA in all substrates. All these amino acid residues (Arg204, Lys210, Trp284, and Ser287) belonged to the C1 subdomain, whereas the other amino acid residues located in the substrate-binding site belonged to the N1 or N2 subdomains (Fig. 2B). Therefore, two sulfate groups in CΔ4S6S shifted the arrangements of amino acid residues in the C1 subdomain, resulting in conformational changes in Smon0123/CΔ4S6S owing to the movement of the C domain. Moreover, Smon0123 showed lower affinity with CΔ4S6S than the other chondroitin disaccharides (CΔ0S, CΔ4S, and CΔ6S), suggesting the influence of the interactions among the four subdomains on the affinity level with substrates.

### Little enhancement of ATPase activity of the ABC transporter by CΔ4S6S-bound Smon0123

**Figure 6:**
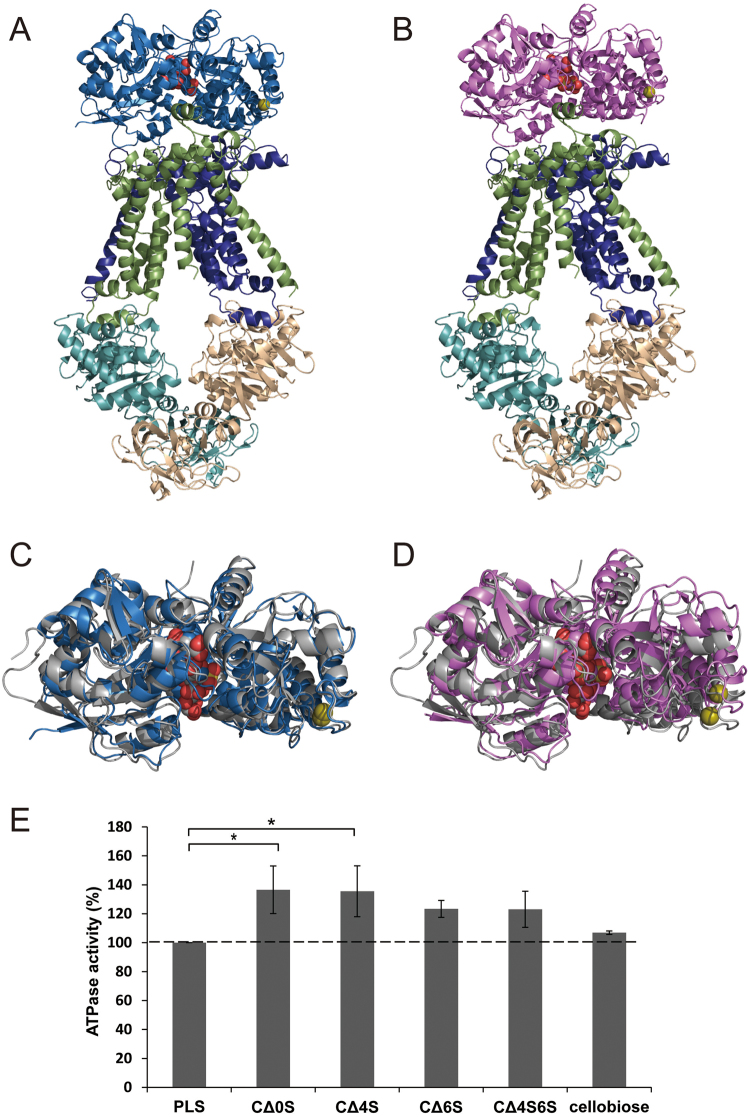
Interaction between Smon0123 and Smon0121-Smon-0122/Smon0120-Smon0120. Structure modeling of the complex of CΔ6S-bound Smon0123 (**A**) or CΔ4S6S-bound Smon0123 (**B**) and Smon0121-Smon0122/Smon0120-Smon0120. Dark blue, Smon0121; light green, Smon0122; cyan and light orange, Smon0120; blue, Smon0123/CΔ6S; and pink, Smon0123/CΔ4S6S. The olive ball shows calcium ion. (**B**) The superimposition of CΔ6S-bound Smon0123 (**C**) or CΔ4S6S-bound Smon0123 (**D**) on AlgQ2 (gray). (**E**) ATPase activity of the Smon0121-Smon0122/Smon0120-Smon0120 in liposomes in the absence of disaccharide (PLS, proteoliposome) or presence of CΔ0S, CΔ4S, CΔ6S, CΔ4S6S, and cellobiose. Each data represents the average of triplicate individual experiments (means ± standard errors of the means). Student’s t-test, *P* > 0.05.

To examine the substrate specificity of the ABC transporter for CΔ4S6S, ATPase activity of the purified Smon0121-Smon0122/Smon0120-Smon0120 reconstructed in liposomes was measured in the presence of Smon0123 and various disaccharides (Fig. 6E). The ATPase activity in the presence of cellobiose as a negative control was comparable with that in the absence of disaccharides (PLS, proteoliposome), while the ATPase activity in the presence of non-sulfated CΔ0S, monosulfated CΔ4S and CΔ6S, and disulfated CΔ4S6S were all enhanced compared to PLS. Although the ATPase activity in the presence of CΔ6S and CΔ4S6S was comparable, the activity in the presence of CΔ4S6S was lowest among these unsaturated chondroitin disaccharides, suggesting that CΔ4S6S bound to Smon0123 in the 39°-closed conformation was possibly unpreferable for ABC transporter in comparison with other GAG disaccharides bound to Smon0123 in the 47°-closed conformation.

## Discussion

Solute-binding proteins were previously classified as six clusters on the basis of the features of their three-dimensional structures^20^. A new 7^th^ cluster has been more recently proposed on the basis of structural features, with relatively a large molecular mass, and EF hand-like calcium-binding sites, which were significantly different from other clusters^21,27^. The members of the 7^th^ cluster contain only four proteins, namely two alginate-binding AlgQ1 and AlgQ2 in *Sphingomonas* sp. A1, uncharacterized Blon_2351 in *Bifidobacterium logum*, and fructooligosaccharide-binding FusA in *Streptococcus pneumoniae*. The EF hand motif, which is the most common calcium-binding motif in proteins, is usually formed by a helix-loop-helix structure. Calcium ion is usually bound by seven ligands at the vertices of a pentagonal bipyramid^28^. Several atypical motifs that vary in length and secondary structure are called EF hand-like motifs^27^. Because of a large molecular size (57 kDa) and EF hand-like calcium-binding site, Smon0123 should also be added to the 7^th^ cluster. Seven oxygen atoms of Asp189, Asn191, Asn193, Lys195, Asp197, and Glu198 of Smon0123 were coordinated with the calcium ion. Although the EF hand-like calcium-binding site had no effect on the substrate binding owing to the remote distance, the motif in the alginate-binding AlgQ2 interacts with the ABC transporter for alginate import in the crystal structure^10^, suggesting its role for substrate translocation from the solute-binding protein to the ABC transporter^27^.

Solute-binding proteins form open and closed conformations in equilibrium even in the absence of substrates^29^. The cleft is opened up to the solvent to allow substrate to freely bind and dissociate, causing flexibility of the structure^30^. Some studies have reported that the crystal structures of substrate-free binding proteins such as leucine-binding protein^29^, allose-binding protein^30^, galactose-binding protein^31^, and ribose-binding protein^32^ form a range of open conformation. However, once the protein recognizes the substrates and closes its domains, the reopening of the protein should be severely restricted because the substrate-bound protein needs to form a complex with the ABC transporter and transfer the substrate. The substrate first binds to a domain of the protein, followed by contact with another domain owing to thermal fluctuations and forms additional contacts to stabilize the closed conformation^19^. Therefore, substrate-bound conformations show much less variations^29^. The glucose-binding protein of *Pseudomonas putida* forms both glucose and galactose-bound structures, and these two complex structures adopt the closed conformation by the hinge-bending motion, although the magnitude of the hinge-bending motions between the two structures are identical^33^. Furthermore, AlgQ1 and AlgQ2, which are homologous to Smon0123, exhibit no structural changes depending on various oligoalginates with different constituent sugars and/or polymerization degrees^34,35^. Conversely, the structures of some solute-binding proteins assumed a different hinge-bending motion even in the presence of same substrates, probably because of the constraints placed on the protein by the crystal-packing interactions^36^. The possibility that the different conformational change between CΔ4S6S-bound Smon0123 and other substrate-bound Smon0123 is owing to crystallization conditions is low because Smon0123/CΔ4S6S did not form crystals under the crystallization conditions of Smon0123/CΔ0S, Smon0123/CΔ4S, and Smon0123/CΔ6S, and vice versa. Furthermore, to clarify this assumption, Smon0123 was again crystallized in the presence of CΔ4S6S with different crystallization conditions for structure determination. The magnitude of the hinge-bending motion in the crystal structure was unexpectedly determined to be 47°; however, C∆4S was bound to Smon0123 instead of CΔ4S6S, which was used as a ligand. This is possibly because of the release of the sulfate group at the C-6 position from CΔ4S6S during crystallization or the presence of contaminated CΔ4S in CΔ4S6S. No sulfatase activity of Smon0123 was also examined by thin-layer chromatography (Fig. S1).

The alginate ABC transporter in *Sphingomonas* sp. A1 comprises four subunits of the transmembrane domain (AlgM1-AlgM2) and ATP-binding domain (AlgS-AlgS). The complex structure of the alginate ABC transporter (AlgM1-AlgM2/AlgS-AlgS) with the alginate-binding protein AlgQ2 indicates the interaction mode between the binding protein and the ABC transporter^10^. In particular, the helix 5c of AlgM2 is crucial for the interaction with AlgQ2. Each subunit of the alginate ABC transporter shows >45% sequence identities with that of the *Streptobacillus* one (AlgM1 vs. Smon0121, 49%; AlgM2 vs. Smon0122, 45%; AlgS vs. Smon0120, 49%; and AlgQ2 vs. Smon0123, 39%). To predict the structure of the *Streptobacillus* ABC transporter, homology modeling was performed using the SWISS-MODEL program^37^ (Fig. 6A and B). Each subunit (Smon0120, Smon0121, and Smon0122) was modeled and merged to the alginate ABC transporter, resulting in a very similar structure. The crystal structures of substrate-bound Smon0123 and AlgQ2 formed complexes with AlgM1-AlgM2/AlgS-AlgS, which were also very well superimposed. Smon0123 probably interacts with the ABC transporter by binding to the helix 5c, also conserved in Smon0122. Dali server^38^ was used to clarify whether Smon0123/CΔ6S or Smon0123/CΔ4S6S is similar to substrate-bound AlgQ2 in the complex form (AlgQ2/AlgM1-AlgM2/AlgS-AlgS). The substrate-bound AlgQ2 showed a higher *Z*-score with Smon0123/CΔ6S (Fig. 6C) than with Smon0123/CΔ4S6S (Fig. 6D), indicating that C∆0S, C∆4S, and C∆6S-bound Smon0123 forms (47°-closed structures) are more suitable than C∆4S6S-bound ones (39°-closed structures) to interact with the ABC transporter (Smon0121-Smon0122/Smon0120-Smon0120). Substrate-bound AlgQ2 in the closed conformation has recently been revealed to induce ATP hydrolysis by interacting with inward-facing AlgM1-AlgM2/AlgS-AlgS^39^. In the case of the CΔ4S6S-bound 39°-closed form, proper docking between the binding protein and ABC transporter and the following ATP hydrolysis and substrate import are considered to be somewhat difficult. We have previously investigated the enzymatic properties of Smon0127 UGL for degrading GAG disaccharides^8^. Smon0127 prefers non-sulfated GAG disaccharides over sulfated GAG disaccharides. Considering the enzymatic properties of Smon0127, the ABC transporter is possibly unable to incorporate CΔ4S6S to save energy generated via ATP hydrolysis. To confirm this hypothesis, we investigated the ATPase activity of the ABC transporter in the presence of Smon0123 and CΔ4S6S (Fig. 6E). As a result, the enhancement of its ATPase activity in the presence of CΔ4S6S was lower than that of CΔ0S and CΔ4S, suggesting that CΔ4S6S-bound Smon0123 in the 39°-closed conformation is unpreferable for docking with the ABC transporter compared to that in the 47°-closed conformation. Therefore, Smon0123 is suggested to select preferable substrates by two steps, based on whether to bind and how to bind. Alternative substrate-bound conformation probably prevents the import of the unfavorable substrate CΔ4S6S.

In conclusion, Smon0123 adopts two 39°- and 47°-closed conformations depending on the sulfate group(s) in the substrate by the hinge-bending motion. The two sulfate groups in CΔ4S6S shift the arrangements of the substrate-binding residues in the C1 subdomain, followed by a dynamic conformational change.

## Materials and Methods

### Materials

CΔ4S6S was purchased from Dextra Laboratories, and chondroitin sulfate A was purchased from Wako Pure Chemical Industries. Columns for metal affinity chromatography (TALON), anion exchange chromatography (Toyopearl DEAE-650M), and gel filtration chromatography (Hi Load 16/60 Superdex 200 pg) were purchased from Clontech, Tosoh Bioscience LLC, and GE Healthcare, respectively. JBScreen Classic 3 for the crystallization kit was purchased from Jena Bioscience. *S. moniliformis* DSM 12112 was purchased from Deutsche Sammlung von Mikroorganismen und Zellkulturen. All other analytical grade chemicals used in this study were commercially available.

### Plate method for detecting GAG degradation

Bacterial cells were cultured on the following medium plates (φ = 90 mm) solidified with 1% agar that contained 1% BSA and 0.2% chondroitin sulfate A, followed by the addition of 1 ml of 2 M acetic acid on the plate. *Pedobacter heparinus* and *Escherichia coli* were used as the positive and negative controls for GAG degradation, respectively. *S. moniliformis* cells were cultured in 0.8% nutrient broth (0.3% beef extract and 0.5% peptone) and 20% horse serum at 37 °C under 5% CO_2_; *P. heparinus* cells were cultured in 0.8% nutrient broth at 30 °C, and *E. coli* cells were cultured in Luria broth (1% tryptone, 0.5% yeast extract, and 1% NaCl)^40^ at 37 °C.

### Protein purification

The overexpression system of Smon0123 has been previously constructed in *E. coli* cells^8^. Full-length Smon0123 was used for the substrate-binding assay by fluorescence spectrum analysis and N and C-terminus-truncated Smon0123 (N-18/C-5) was used for crystallization. Smon0123 was purified from the *E. coli* cell extract using metal affinity chromatography (TALON), anion exchange chromatography (Resource Q), and gel filtration chromatography (Hi Load 16/60 Superdex 200 pg). Anion exchange chromatography (Toyopearl DEAE-650M) and gel filtration chromatography (Hi Load 16/60 Superdex 200 pg) were used to purify Smon0123 (N-18/C-5). Briefly, the ABC transporter (Smon0121-Smon0122/Smon0120-Smon0120) containing His-tagged sequence in the C-terminus of Smon0122 was purified from recombinant *E. coli* cell membrane solubilized with the detergent using metal affinity chromatography [Ni-NTA (Qiagen)] and gel filtration chromatography (Hi Load 16/60 Superdex 200 pg).

### Fluorescence spectrum analysis

To investigate the binding affinity of Smon0123 with CΔ4S6S, the fluorescence intensity of Smon0123 in the presence of CΔ4S6S was measured using a spectrofluorometer [FP-6500 (JASCO)]. CΔ4S6S was added step-by-step (0–20 μM) to the purified 0.1 μM Smon0123. The measurement parameters were as follows: excitation band width, 1 nm; emission band width, 10 nm; response, 2 s; sensitivity, high; excitation wavelength, 280 nm; start to end emission wavelength, 300–500 nm; data pitch, 1 nm; and scan speed, 100 nm/min. The ratio of change in fluorescence intensity in the presence of CΔ4S6S compared with that in the absence of CΔ4S6S was plotted, and the dissociation constant (*K*_d_ value) was determined.

### X-ray crystallography

The purified Smon0123 (N-18/C-5) was crystallized by sitting drop vapor diffusion. In 96-well sitting drop plates, 1 μl of 10.8 mg/ml Smon0123 (N-18/C-5) and an equal volume of reservoir solution for crystallization were mixed in the presence of 0.5 mM CΔ4S6S. The solution was kept at 20 °C for 2 weeks until the crystals sufficiently grew. A single crystal was picked up using a nylon loop from the drop, soaked in a reservoir solution that contained 20% ethylene glycol, and instantly frozen using cold nitrogen gas. Synchrotron radiation X-ray irradiated the crystal at 1.00 Å wavelength, and X-ray diffraction data were collected using the MAR225HE (Rayonix) detector at BL-26B1 beamline in SPring-8 (Harima, Japan). The obtained data were indexed, integrated, and scaled using the *HKL-2000* program^41^. Some crystals in the reservoir solution that contained 18% (w/v) PEG 4000, 20% (w/v) isopropanol, and 0.1 M Na HEPES (pH 7.5) from the JBScreen Classic 3 crystallization kit gave high-resolution diffraction data. The structure was determined through molecular replacement by *Molrep* in the *CCP4* program^42^, with Smon0123/CΔ0S (PDB ID, 5GUB) as the search model. The *PHENIX* program was used to refine the structure; the *winCoot* program^43^ allowed model adjustment. Figures for the protein structure were drawn using the *PyMol* program^44^. Another crystal of Smon0123 in the presence of C∆4S6S was obtained in the droplet consisting of 0.2 M sodium iodide, 0.1 M Bis Tris propane (pH 7.5), and 20% (w/v) PEG 3350, and diffracted to 2.50 Å resolution at BL-38B1 beamline in SPring-8. The crystal belongs to a space group *P*2_1_ and has unit lattice constants of a = 57.8, b = 97.1, c = 79.5 Å, α = 90.0, β = 106, and γ = 90.0°. Similarly, its crystal structure was determined through molecular replacement, although the protein was found to interact with C∆4S but not C∆4S6S in the cleft.

### Magnitude of hinge-bending motion

To calculate the magnitude of the hinge-bending motion, N domains of substrate-free and substrate-bound Smon0123 were superimposed. By using FIT program, rotation matrix, translation vectors, rotation angles, and screwing distances of C domains of substrate-free and substrate-bound Smon0123 were calculated from centers of gravity.

### ATPase assay

As previously described^8^, ATPase activity of Smon0121-Smon0122/Smon0120-Smon0120 reconstructed in phospholipid liposomes was measured in absorbance at 850 nm by the molybdenum blue method to determine phosphate ion concentration generated through ATP hydrolysis in the presence of Smon0123 and ligands.

### Data availability

The coordinates and structure factors of Smon0123/CΔ4S6S have been deposited in the Protein Data Bank (PDB ID, 5XS8).

## Electronic supplementary material

Supplementary Information
