# Supplementary material for: Alternative substrate-bound conformation of bacterial solute-binding protein involved in the import of mammalian host glycosaminoglycans
Source: Sci Rep. 2017 Dec 5;7:17005. doi: 10.1038/s41598-017-16801-8 (PMC5717064; doi:10.1038/s41598-017-16801-8)

Supplementary Information

Alternative substrate-bound conformation of bacterial solute-binding protein involved in the import of mammalian host glycosaminoglycans

Sayoko Oiki[1], Reiko Kamochi[2], Bunzo Mikami[3], Kousaku Murata[4] & Wataru Hashimoto[1, 2]

[1]Laboratory of Basic and Applied Molecular Biotechnology, Division of Food Science and Biotechnology, Graduate School of Agriculture, Kyoto University, Uji, Kyoto 611-0011, Japan; [2]Laboratory of Basic and Applied Molecular Biotechnology, Department of Food Science and Biotechnology, Faculty of Agriculture, Kyoto University, Uji, Kyoto 611-0011, Japan; [3]Laboratory of Applied Structural Biology, Division of Applied Life Sciences, Graduate School of Agriculture, Kyoto University, Uji, Kyoto 611-0011, Japan; [4]Laboratory of Food Microbiology, Department of Life Science, Faculty of Science and Engineering, Setsunan University, Neyagawa, Osaka 572-8508, Japan. Correspondence and requests for materials should be addressed to W. H. (email: whasimot@kais.kyoto-u.ac.jp)

Table S1. Statistics of CΔ4S6S-bound Smon0123 for data collection and structure refinement

	**Smon0123 (N-18/C-5)/CΔ4S6S**
**Data collection**	
Space group	*P*1
Cell dimensions	
a, b, c (Å)	49.7, 69.2, 166
α, β, γ (°)	89.9, 90.0, 90.0
Resolution (Å)	50.0-1.96 (1.99-1.96) *
R_{merge}	7.3 (11.2)
I / σ (I)	28.1 (2.9)
Completeness (%)	97.4 (91.3)
Redundancy	2.1 (1.0)
**Refinement**	
Resolution (Å)	40.4-1.95 (1.97-1.95)
No. reflections	156219 (4217)
R_{work} / R_{free}	17.3 (22.0) / 21.6 (26.9)
No. atoms	
Protein	15332
Sugar	136
Ca^{2+}	4
Water	1293
B-factor (Å^2)	
Protein	24.4
Sugar	21.8
Ca^{2+}	27.1
Water	26.6
R. m. s. deviations	
Bond lengths (Å)	0.02
Bond Angles (°)	1.80
Ramachandran plot (%)	
Favored region	97.9
Allowed region	2.12
Outlier region	0

*Data for the highest resolution shell is shown in parenthesis.

Figure S1. Sulfatase assay. To investigate the sulfatase activity of Smon0123, the reaction mixture containing 70 μM the purified Smon0123, 4 mM CΔ4S, CΔ6S, or CΔ4S6S, and 50 mM Tris-HCl (pH 7.5) was incubated at 30°C for 8 h. The mixture was boiled to stop reaction and centrifuged at 8,000 *g* for 3 min. The resultant supernatants (5 μl) were subjected to thin-layer chromatography (TLC). Lanes 1, Smon0123; 2, Smon0123 and CΔ4S; 3, CΔ4S; 4, Smon0123 and CΔ6S; 5, CΔ6S; 6, Smon0123 and CΔ4S6S; 7, CΔ4S6S; and 8, CΔ0S. As a result, no desulfated product CΔ0S was generated from CΔ4S or CΔ6S by Smon0123 and, similarly, no product, i.e. CΔ0S, CΔ4S, or CΔ6S, was generated from CΔ4S6S by Smon0123, indicating that Smon0123 exhibited no sulfate activity. This profile is not an image cropped from different parts of the same TLC plate or from different TLC plates.

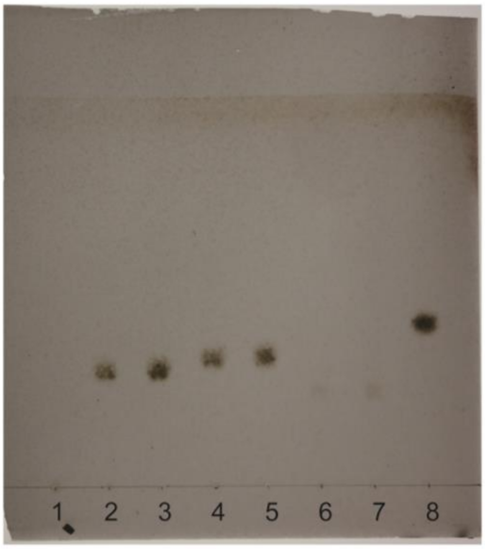

Supplement: Supplementary file 1 — Supplementary Information [file 41598_2017_16801_MOESM1_ESM.pdf]
